# Eliminating the myth of individual penalties in environmental law: Individual penalties and pollution emission reduction before and after the revision of China’s environmental protection act

**DOI:** 10.1371/journal.pone.0319410

**Published:** 2025-04-25

**Authors:** Yuan Jiang, Siying Wang, Xuan Yu, Yi Xiao

**Affiliations:** 1 Law School of Ningbo University, Ningbo, China; 2 Business School of Ningbo University, Ningbo, China; 3 School of Accounting, Zhejiang University of Finance and Economics, Hangzhou, China; Universitas Airlangga, INDONESIA

## Abstract

Developing countries, including China, have long focused on punishing individuals through environmental laws. This focus stems from the fact that individual penalties are increasing and pollution emissions are decreasing. However, the over-emphasis on individual punishment in environmental laws is actually a false superstition. This paper takes the 2015 revision of China’s Environmental Protection Law as the node, analyses the impact of the law revision on the intensity of individual punishment and the degree of pollution emission, and then analyses the correlation between individual punishment and pollution emission reduction. The conclusion is that there is no correlation between individual punishment and pollution emission reduction. As a result, this paper dispels the myth of individual punishment in environmental laws. With the rapid development of economy and society, China’s environmental law should stimulate enterprises, citizens and public welfare organisations to participate, so as to change the purely punitive governance into collaborative governance.

## 1 Introduction

The problem of environmental pollution stems from the fact that individuals have discharged too many pollutants, resulting in the discharge of pollution that exceeds the carrying capacity of the ecological environment [1]. Therefore, the initial environmental laws were concerned with punishing individuals for their illegal discharge of pollutants. The purpose of punishing individuals in environmental laws is not limited to the punishment itself, but to deter potential dischargers from discharging pollutants in violation of the law. However, environmental laws that focus solely on punishing individuals may encounter a series of problems, including but not limited to regulatory capture and rent-seeking. In China, Regulatory Capture is generally manifested as illegal state, that is, the corruption of administrative bureaucrats. Individuals bribe environmental law enforcement officials in various forms, thereby exempting them from punishment while emitting pollution, or only receiving smaller punishments [2]. However, rent-seeking is generally in a legal state, that is, local governments may form an interest exchange relationship with individuals who discharge pollution. Individuals obtain the right to discharge pollution in disguise by accepting corresponding punishments, while local governments obtain corresponding political achievements by punishing individuals. Both of them can gain more attention to their own interests, which in turn leads to increased environmental pollution. No matter which of the two, the final result is that punishment cannot effectively reduce environmental pollution [3]. Therefore, the environmental laws of many modern developed countries have undergone a transformation that goes beyond punishing individuals. The transformation is mainly manifested in two aspects: firstly, environmental laws are no longer limited to punishment as a form of regulation [4], but rather use property rights and market-based means to motivate individuals to avoid, as far as possible, polluting and emitting behaviours that have no added value or little added value. Secondly, environmental law no longer focuses only on the individual, but includes the government in the scope of regulation, thus requiring the government to carry out collaborative governance [5].

However, this transition is not applicable to all countries, especially developing countries with relatively low economic levels. The use of property rights and market-based instruments in environmental law requires both more additional work to address boundary rights and circulation costs, and a wider field for market circulation, which means it takes up more economic and rule of law costs [6]. Furthermore, for governments in developing states, Collaborative governance likewise entails societal costs that are hard for them to bear. [7] Therefore, environmental laws that emphasise the punishment of individuals may be a more appropriate choice. On the one hand, the various costs of punishing individuals are relatively low and can be spread to all the subjects of the whole legal system, rather than having to bear the social costs alone; on the other hand, in the primary stage of the environmental law, punishing individuals can indeed produce a certain pollution abatement effect, and the effect is produced relatively quickly [8].

The problem becomes more complex for countries that are developing very rapidly and are in the process of transitioning from developing to developed countries. These countries often have environmental laws that emphasise individual penalties, and have identified a range of problems that arise with the punishment of individuals, but are not willing to break through individual penalties to reform their environmental laws [9]. One important reason is that in the legal practice of these countries, environmental laws that emphasise individual penalties have indeed achieved significant pollution reduction [10]. Furthermore, these countries firmly believe that punishing individuals should be the core of environmental laws, and that the problems associated with individual punishment are the deficiencies in the technical means of regulation and punishment, rather than the defects in the origin of punishing individuals [11]. A typical example of such countries is China.

However, the simultaneous existence of individual penalties and the effectiveness of pollution abatement in space and time does not mean that there is a sufficient causal link between the two. The myth of individual penalties as a sufficient justification for pollution abatement, and the resulting fetishisation of individual penalties, is a trap that has prevented the reform of environmental laws in these countries.

This paper attempts to take China’s Environmental Protection Law as an object and its 2015 revision as a node, and by analysing the correlation between the variable of the intensity of punishing individuals and the variable of the degree of pollution emission before and after its revision, it proves that there is no obvious correlation between individual punishment and pollution reduction. The marginal contributions of this paper are: (1) to prove that “the enhancement of individual penalties cannot necessarily reduce pollution emissions”, analyse the reasons for this, and then dispel the superstition of environmental laws on individual penalties, and provide key support for the legal transformation beyond individual. penalties. (2) Evaluating the strength of environmental laws on individual regulation from a new perspective. Many studies have directly taken the degree of pollution emission or the frequency of environmental keywords in government reports as the regulatory intensity of environmental laws [12]. In fact, the degree of pollution emission is a regulatory outcome of environmental laws, while individual punishment is a regulatory means of environmental laws, the latter is a new perspective of regulatory assessment. (3) To explore environmental jurisprudence in a relatively novel way, the usual methods of legal research are textual analysis and case study analysis, and the quantitative analysis adopted in this paper is an extremely meaningful attempt.

The structure of this paper is divided into three parts: the first part provides a background introduction to the state of environmental rule of law in China and the amendment of the Environmental Protection Law (EPL) in 2015; the second part analyses the impact of the amendment of the EPL on the penalty intensity and pollution emission reduction, as well as analyses the correlation between the penalty intensity and pollution emission reduction; and the third part discusses and concludes the empirical results.

## 2 Background and assumptions

### 2.1 Environmental laws in China

In order to dispel the myth of individual penalties in environmental laws, it is necessary to test the correlation between individual penalties and pollution abatement through an experiment on environmental laws in a certain country.That is to test whether individual punishment can reduce pollution emissions in the short or long term. In order to test the accuracy and rigour of the experiment, the experiment has more stringent requirements on the sample. Firstly, the country must be in the stage of transition from developing to developed country, with sufficient economic foundation and governance capacity. The reform of environmental laws beyond individual punishment requires sufficient economic foundation and governance capacity to support, and countries that do not have both lack the need to conduct the experiment. Second, the country must attach great importance to environmental law and be adept at using individual penalties to combat environmental pollution in legal practice. If the country’s environmental laws are not centred on individual penalties, then individual penalties are unlikely to have a decisive impact on pollution emissions, and the two cannot be put into a unified test in the experiment. The purpose of the experiment is to examine whether there is a correlation between individual penalties and reductions in total pollution. Only environmental laws built around individual penalties are eligible for the experiment. Only legal practices that strictly implement individual penalties can link individual penalties and pollution reduction in the experiment.

Compared to the above requirements, China is a near-perfect experimental sample for the following reasons.

China is in a period of transition from a developing to a developed country, and has considerable economic fundamentals and governance capacity. Since China’s reform and opening up, China’s economy has grown rapidly and has become the second largest country in the world in terms of total GDP [13]. With sufficient economic foundation as a strong backing for the implementation of laws, the Chinese government has not been stingy in investing capital in the construction and implementation of laws. Through continuous exploration and development, the large amount of economic investment has gradually transformed into the Chinese government’s progress in environmental law and the development of environmental governance [14]. By now, China has the economic foundation and governance capacity to reform environmental governance, and has entered a stage where environmental law should be transformed.

China attaches great importance to environmental law and the rule of law. As early as the 1980s, environmental protection was written into China’s basic State policy. Over the years, leaders of the central government have repeatedly emphasised that “the strictest rule of law will be used to combat environmental pollution”, and have continued to revise and add various types of environmental laws [15]. In China’s overall approach to environmental pollution control, environmental law has become the leader of other governance tools, and has been consolidated and implemented through the expression “pollution control according to law” [16]. Accordingly, China has achieved remarkable results in reducing pollution. According to the 2022 China Environmental Statistics Yearbook, emissions of all types of pollutants are at or near historic lows since the turn of the century, and emissions reductions in both air and water pollution have exceeded targets. In addition, China’s environmental laws are centred on punishing individuals, and in legal practice they place great emphasis on punishing individuals [17]. In terms of legal settings, all Chinese environmental laws are divided into three parts. The General Provisions section, which functions as a declaration of principles and does not contain specific regulations. The rules section, with the individual as the main object of regulation, is mainly composed of prohibitive or standard clauses, and its function is to set up rules of behaviour for the individual. The penalty part, with the individual as the main object of regulation, mainly consists of punitive clauses, the function is to provide for the punishment of the individual after violating the rules. The Environmental Protection Law, the Water Pollution Prevention and Control Law, the Air Pollution Prevention and Control Law, and the Marine Environmental Protection Law are all set up in this way. From this, it can be seen that China’s environmental laws are laws that focus on individual punishment. In legal practice, the Chinese government not only carries out regular law enforcement and judicial activities in accordance with the setting of environmental laws, but also continuously strengthens the punishment of individuals by means of campaign-style governance. A typical example of this is the environmental protection inspections that began in 2015. It has been conducting nationwide screening for several years, with the main task of imposing heavier penalties on a large number of individuals who have violated the law [18]. However, environmental laws emphasize that the punishment of individuals is not always effective. It may be due to many reasons that environmental violations cannot be effectively found, or the punishment for environmental violations is insufficient. These include limited administrative costs, some systematic failures or advancement of technology, etc.The punishment failure caused by these reasons cannot be solved by simply increasing the punishment. In addition to the ability and efficiency of punishment itself, there are other factors that may lead to the fact that the law that simply punishes individuals cannot completely solve environmental problems. Many studies have shown that even when environmental violations are effectively regulated and punished, local environmental quality may still decline [19]. Especially when the rent-seeking relationship is formed between some industrial interest groups that cause serious pollution and local governments, the punishment of individuals often appears to be weak. For example, a large number of state-owned enterprises in China have caused serious environmental pollution, but environmental laws cannot effectively inhibit these pollutions. The pollution case of Zijin Mining, which shocked the whole of China, illustrates this point well. As a large state-owned enterprise, Zijin Mining Group caused direct economic losses of 100 million yuan ($ 14 million) in 2010 due to pollution emissions, which did not calculate the costs related to follow-up treatment and ecological restoration. However, according to the environmental protection law, only a fine of 30 million yuan ($ 4.2 million) was imposed on Zijin Mining [20].

### 2.2 Environmental protection act as amended in 2015

In China’s environmental legal system, the Environmental Protection Law is in the position of the basic law. The Environmental Protection Law was chosen as the object of study both because it represents well all environmental laws in China and because its legislative direction determines the legislative direction of other environmental laws.

The revised Environmental Protection Law of 2015 has been called the “toughest” environmental protection law in history, which not only focuses on behaviours that have already resulted in pollution, but also requires that behaviours that have not, but may, result in pollution be regulated [21]. Compared to all previous environmental laws, the revised EPL strengthens individual penalties in four ways. First, the regulatory system is more comprehensive, such as combining regional EIA with construction EIA, combining environmental standards with total volume control, and combining permit management with environmental credit. The second is tougher regulatory tools, such as stipulating that local governments can set stricter standards for individual discharges, granting law enforcement authorities the right to seize and impound, and providing for a system of over-the-top reporting. Thirdly, the administrative penalties are more severe, such as raising the penalty standards, providing for a daily penalty system, establishing joint and several liability for the environment, and providing for administrative detention as a personal coercive measure. Fourth, the supervision mechanism is more powerful, such as the provisions of the administrative assessment mechanism, the establishment of the National People’s Congress supervision mechanism [22].

Following the 2015 amendments to the Environmental Protection Act (EPA), many studies examining the regulatory intensity of the EPA have concluded that the amendments have further strengthened the regulatory intensity of the EPA [23]. Although these studies do not limit the regulatory intensity of the EPA to the punishment of individuals, we can deduce from the setting and implementation of the EPA that the revision strengthened the regulatory intensity of the EPA mainly in terms of individual punishment. In the following, this paper will take a new approach to make a more accurate assessment of the individual punishment intensity of the Environmental Protection Law.

### 2.3 Verifiable hypotheses

This paper will first test whether the 2015 amendment to the Environmental Protection Law has increased individual punishment intensity and reduced pollution emissions, thus providing the basis for the subsequent test: a scenario of increasing punishment intensity and decreasing pollution emissions. Then, this paper will test whether there is a correlation between punishment intensity and pollution emission reduction, so as to prove that punishing individuals is not a sufficient condition for pollution emission reduction, and thus dispel the myth of individual punishment in environmental laws. The experimental samples selected in this paper are the individual punishment intensity and pollution emission level before and after the revision of the Environmental Protection Law in 2015, on which this paper puts forward three hypotheses that can be tested in turn.These three hypotheses are derived from scholars ’ questioning of the effectiveness of China ’s strict environmental protection law : enhanced individual punishment does not absolutely reduce pollution emissions.

H1: After the revision, environmental laws penalise individuals significantly more.
H2: The extent of polluting emissions is significantly reduced after the revision.
H3: There is no correlation between the enhancement of individual penalties and the reduction of pollution emissions.

## 3 Research methodology

To test the three hypotheses presented, the following three regression models are set up in this paper:


$$\begin{array}{ccc} {intensity}_{it}=\beta_{1}D_{it}+\beta_{2}control_{it}+{𝜀}_{it}+u_{s} & & \end{array}$$ (1)

$$\begin{array}{ccc} EP_{it}=\beta_{1}D_{it}+\beta_{2}control_{it}+{𝜀}_{it}+u_{s} & & \end{array}$$ (2)

$$\begin{array}{ccc} EP_{it}=\beta_{1}intensity_{it}+\beta_{2}control_{it}+{𝜀}_{it}+u_{s}+\gamma_{t} & & \end{array}$$ (3)

Among them, Models 1 and 2 are used to examine the effects of the amendment of the Environmental Protection Law on the intensity of individual penalties and the degree of pollution emission, respectively; Model 3 examines the effects of the intensity of individual penalties on the degree of pollution emission. In the model, i is the region, which includes data from 30 provinces in China, excluding Tibet, Hong Kong, Macao and Taiwan due to data availability. t is the time, which spans from 2009 to 2021. EP is the degree of pollution emission, and the specific variables include the two most common environmental pollution factors: industrial fume (dust) emissions and sulfur dioxide emissions. intensity is the intensity of individual punishment, which includes the intensity of environmental protection administrative punishment and the intensity of environmental protection penalty. Including environmental protection administrative penalty intensity and environmental protection judicial intensity of two categories, respectively, the number of environmental protection administrative penalty cases per unit of enterprise and the number of environmental protection judicial cases per unit of enterprise as a characterisation. d is a dummy variable for determining whether it is 01 after the revision of the Environmental Protection Law, with the revision of the Environmental Protection Law in 2015 as the node. control is a series of control variables, $u_{s}$ for the individual fixed effects, $\gamma_{t}$ for the time fixed effect, ${𝜀}_{it}$ is the error term. In the above data, except for the number of enterprise environmental protection administrative penalty cases and the number of enterprise environmental protection judicial cases from the authoritative rule of law data public website - China Judicial Judgement Network, the remaining data come from the China Environmental Statistics Yearbook from 2009–2021.

For the selection of control variables, based on the STIRPAT model, economic level, population density, and scientific and technological level were selected as control variables [24]. The economic level is characterised by GDP per capita, population density by population per square kilometre, and the number of patent applications granted by the number of patent applications granted, and all the data are obtained from the China Statistical Yearbook from 2009 to 2021.The reason for choosing these three data sources is that the punishment of individuals by law is generally divided into law enforcement and justice. The number of corporate environmental protection administrative penalty cases well represents the government ’s environmental law enforcement intensity to individuals, and the number of corporate environmental protection judicial cases well represents the court ’s environmental justice intensity to individuals.Environmental law enforcement is carried out by the government ’s environmental protection administrative department, so it can be clearly separated from other administrative penalty cases. Like many developing countries, China has set up special environmental courts [25]. Therefore, environmental protection cases can be clearly distinguished from other departmental law cases. The combination of the two can well represent the intensity of individual punishment. The data of pollution emissions can directly reflect the change of pollution control level. The data are separately and clearly listed in the ’ China Statistical Yearbook ’.

Based on the EKC hypothesis, the inverted U-shaped relationship that exists between economic level and environmental pollution [26], so the squared term of economic level is added to the control variables. In addition, in order to eliminate the effect of heteroskedasticity, all absolute value variables are made logarithmic, and the specific meaning of each variable is shown in Table 1.

**Table 1:** Table of meanings of variables.

Variable type	Variable Meaning	Specific indicators	Variable Abbreviations	Data sources
Explained Variables and Explanatory Variables	Degree of pollution emission	Industrial fume (dust) emissions (logarithmic)	lnsmoke	China Environmental Statistics Yearbook (2010–2022)
		Sulphur dioxide emissions (logarithmic)	lnsulfur	
	Intensity of environmental administrative penalties	Number of environmental protection administrative cases/number of enterprises	penalty	
	Environmental justice intensity	Number of environmental justice cases/number of enterprises	judicial	
	Amendments to the Environmental Protection Act	Yes = 1, No = 0	D	
control variable	economic level	GDP per capita (logarithmic)	lnpgdp	China Statistical Yearbook (2010–2022)
		GDP per capita (logarithmic) squared term	lnpgdp2	
	population density	Population per square kilometre (logarithmic)	lnpop	
	technological level	Number of patent applications granted (logarithmic)	lntech	

Descriptive statistics of all sample data are shown in Table 2.

**Table 2:** Descriptive statistics of data.

Variable	N	Mean	SD	Min	Max
lnsmoke	390	12.30	1.019	8.362	14.40
lnsulfur	390	12.28	1.454	6.215	14.95
lnpgdp	390	10.78	0.505	9.241	12.12
lntech	390	10.02	1.523	5.576	13.68
lnpop	390	5.463	1.292	2.043	8.282
lnpgdp2	390	116.4	10.90	85.39	147.0
judicial	390	0.580	0.907	0.000252	8.424
penalty	390	0.521	1.063	0.000850	9.701
d	390	0.538	0.499	0	1

## 4 Empirical results

### 4.1 Benchmark regression results

Table 3 reports the estimation results of Model 1, which assesses the impact of the EPA amendments on the intensity of individual penalties. In this case, columns (1) and (2) do not include control variables, while columns (3) and (4) show the results after the inclusion of control variables. The estimation results show that the F-tests of the four sets of regression models passed the 1% significance test, indicating that the joint significance of the variables in the models is high and the regression results have a high degree of confidence.

**Table 3:** Estimated results of the impact of EPA amendments on individual penalties.

	(1) judicial	(2) penalty	(3) judicial	(4) penalty
D	0.8105^***^ (0.0606)	0.0709 (0.0975)	0.5807^***^ (0.0959)	–0.2536 (0.2165)
_cons	0.0762 (0.0744)	0.6843^**^ (0.3298)	–21.4809 (21.7042)	–63.1792^*^ (38.0698)
control variable	No	No	Yes	Yes
fixed time	No	No	No	No
fixed ind	Yes	Yes	Yes	Yes
F-test	10.81^***^	8.23^***^	14.98^***^	3.86^***^
R^2^	0.5833	0.2878	0.5946	0.3452
N	390	390	390	390

Robust standard errors in parentheses ^*^ p < 0.1,^**^p < 0.05,^***^p < 0.01

From the results of coefficient estimation of variable D in Table 3, it can be found that when environmental protection judicial intensity (judicial) is used as an explanatory variable, the estimated coefficient of the core explanatory variable D is significantly positive at the 1% level, regardless of whether or not control variables are added. However, when environmental protection administrative penalty intensity (penalties) is used as an explanatory variable, the estimated coefficients of the core explanatory variable D do not pass the significance test, regardless of whether control variables are added or not. The above results indicate that the amendment of the Environmental Protection Law in 2015 significantly increased the intensity of environmental protection justice, but did not have a significant effect on the intensity of environmental protection administrative penalties, and Hypothesis 1 is partially proved.

Table 4 further reports the estimation results of Model 2, which assesses the impact of the amendments to the Environmental Protection Act 2015 on the level of environmental pollution emissions. In this case, columns (1) and (2) do not incorporate control variables, while columns (3) and (4) show the results after incorporating control variables. The estimation results show that the F-tests of the four sets of regression models pass the 1% significance test, indicating that the joint significance of the variables in the models is high and the regression results have a high degree of confidence. In addition, the results of the R^2^ of the four groups of models also indicate that the variables selected in the models are able to highly fit the changes in the explanatory variables.

**Table 4:** Results of the estimation of the impact of the amendments to the environmental protection act on pollution discharges.

	(1) lnsmoke	(2) lnsulfur	(3) lnsmoke	(4) lnsulfur
D	–0.4052^***^ (0.0344)	–1.4860^***^ (0.0794)	–0.4218^***^ (0.0616)	–0.2479^**^ (0.1067)
_cons	11.1457^***^ (0.1567)	11.3730^***^ (0.3959)	–19.9223^*^ (10.7832)	–204.1064^***^ (20.9264)
control variable	No	No	Yes	Yes
fixed time	No	No	No	No
fixed ind	Yes	Yes	Yes	Yes
F-test	90.17^***^	33.32^***^	84.87^***^	128.91^***^
R^2^	0.8958	0.6989	0.9007	0.8459
*N*	390	390	390	390

Robust standard errors in parentheses ^*^ p < 0.1,^**^p < 0.05,^***^p < 0.01

From the coefficient estimation results of variable D in Table 4, it can be found that when industrial smoke (powder) dust emissions (lnsmoke) and sulphur dioxide emissions (lnsulfur) are used as explanatory variables, the estimated coefficients of the core explanatory variable D are all significantly negative at the 1% level, regardless of whether control variables are added or not. This result indicates that the amendment of the Environmental Protection Act in 2015 significantly reduces industrial lnsmoke and lnsulfur dioxide emissions, i.e., the amendment of the Environmental Protection Act significantly reduces pollution emissions, and Hypothesis 2 is proved.

Table 5 reports the estimation results of model 3, which assesses the impact of individual penalties on pollution emissions. Columns (1) and (2) examine the effect of the intensity of environmental administrative penalties (penalties) on pollution emissions, while columns (3) and (4) examine the effect of the intensity of environmental judicial (judicial) on pollution emissions. The estimation results reported in Table 5 show that the F-tests of the four sets of regression models pass the 1% significance test, indicating that the joint significance of the variables in the models is high and the regression results have a high degree of confidence. In addition, the results of the R2 of the four groups of models also indicate that the variables selected in the models are able to highly fit the changes in the explanatory variables, with goodness of fit of 93.91%, 89.46%, 93.92% and 89.45%, respectively.

**Table 5:** Estimated results of the impact of individual penalties on pollution emissions.

	(1) lnsmoke	(2) lnsulfur	(1) lnsmoke	(2) lnsulfur
penalty	0.0293 (0.0241)	–0.0277 (0.0237)		
judicial			0.0479 (0.0366)	–0.0293 (0.0326)
_cons	–16.6981 (10.9800)	–108.2025^***^ (18.1008)	–15.4939 (11.4384)	–108.9188^***^ (18.1238)
control variable	Yes	Yes	Yes	Yes
fixed time	Yes	Yes	Yes	Yes
fixed ind	Yes	Yes	Yes	Yes
F-test	151.61^***^	174.21^***^	168.85^***^	139.81^***^
R^2^	0.9391	0.8946	0.9392	0.8945
N	390	390	390	390

Robust standard errors in parentheses ^*^ p < 0.1,^**^p < 0.05,^***^p < 0.01

From the coefficient estimation results of each variable in Table 5, it can be found that: whether industrial smoke (dust) emissions (lnsmoke) or sulfur dioxide emissions (lnsulfur) as the explanatory variables, the core explanatory variables of the intensity of environmental protection administrative penalties (penalty) and environmental protection judicial intensity (judicial) of the coefficient estimation in the statistical significance of the coefficient did not pass the test of significance. . The above regression results indicate that the intensity of environmental individual penalties in the region will not have a significant effect on the pollution emissions in the region, and Hypothesis 3 is proved.

### 4.2 Robustness tests

#### 4.2.1 Replacing explanatory variables.

In order to test the robustness of the above benchmark regression results, and considering the possible lag in the impact of policy effects [27], i.e., the penalties imposed on individuals may act on future pollution emissions rather than reacting in time to the pollution emissions in the same year, this paper re-estimates the model by replacing all explanatory variables in the benchmark regression model (1)–(3) by their lagged one-period counterparts. The estimation results are shown in Tables 6 and 7.

**Table 6:** Estimates with $D_{t-1}$ as explanatory variable.

	(1) judicial	(2) penalty	(3) lnsmoke	(4) lnsulfur
Dt-1	0.4853^***^ (0.1035)	–0.0361 (0.1837)	–0.6936^***^ (0.0492)	–0.6514^***^ (0.1021)
_cons	–25.0430 (26.2697)	–76.8773^*^ (46.3418)	–1.0168 (11.0653)	–211.3895^***^ (23.0955)
control variable	Yes	Yes	Yes	Yes
fixed time	No	No	No	No
fixed ind	Yes	Yes	Yes	Yes
F-test	13.09^***^	3.85^***^	104.22^***^	239.18^***^
R^2^	0.6054	0.3772	0.9335	0.8697
N	360	360	360	360

Robust standard errors in parentheses ^*^ p < 0.1,^**^p < 0.05,^***^p < 0.01

**Table 7:** Estimates with $penalty_{t-1}$ and $judicial_{t-1}$ as explanatory variables.

	(1) lnsmoke	(2) lnsulfur	(3) lnsmoke	(4) lnsulfur
penalty t-1	0.0207 (0.0210)	–0.0296 (0.0232)		
judicial t-1			0.0550 (0.0331)	0.0050 (0.0295)
_cons	–7.2915 (12.0068)	–131.6852*** (20.6955)	–6.2767 (12.4087)	–131.7650*** (20.7734)
control variable	Yes	Yes	Yes	Yes
fixed time	Yes	Yes	Yes	Yes
fixed ind	Yes	Yes	Yes	Yes
F-test	150.6***	171.3***	157.83***	214.81***
R^2^	0.9497	0.8960	0.9502	0.8957
*N*	360	360	360	360

Robust standard errors in parentheses * p < 0.1,**p < 0.05,***p < 0.01

The results reported in Table 6 show that the estimated. coefficients of the core explanatory variable $D_{t-1}$ on judiciary are significantly positive at the 1% level, and the estimated coefficients on penalties do not pass the significance test. In addition, the estimated coefficients of the core explanatory variable $D_{t-1}$ or both lnsmoke and lnsulfur are significantly negative at the 1% level.The results reported in Table 7 also show that the estimated coefficients on the effects of the core explanatory variables $penalty_{t-1}$ and $judicial_{t-1}$ on lnsmoke and lnsulfur both fail the significance test, indicating that the intensity of individual penalties in the previous period also does not significantly affect the level of pollution emissions in the current period.

#### 4.2.2 Changing the sample

Considering that the sample contains two types of administrative divisions, municipalities directly under the central government and provinces, these two types of provincial administrative divisions in China differ in various aspects such as legal regulation and pollution control [28]. In order to address the possible estimation bias, this paper further excludes the municipality samples from the sample, specifically including the exclusion of the samples from the four municipalities of Shanghai, Beijing, Tianjin, and Chongqing. Tables 8 and 9 report the results after re-estimation.

**Table 8:** Estimated results of the impact of the amendments to the environmental protection law on individual penalties and pollution emissions (excluding the sample of municipalities).

	(1) judicial	(2) penalty	(3) lnsmoke	(4) lnsulfur
D	0.6032^***^ (0.1022)	–0.2226 (0.2307)	–0.4080^***^ (0.0615)	–0.2242^*^ (0.1159)
_cons	–61.2355^***^ (16.8452)	–67.4535^**^ (26.5286)	–7.1077 (9.2400)	–158.4407^***^ (25.2210)
control variable	Yes	Yes	Yes	Yes
fixed time	No	No	No	No
fixed ind	Yes	Yes	Yes	Yes
F-test	16.34^***^	3.83^***^	76.01^***^	108.02^***^
R^2^	0.5880	0.2738	0.8692	0.7833
*N*	338	338	338	338

Robust standard errors in parentheses ^*^ p < 0.1,^**^p < 0.05,^***^p < 0.01

**Table 9:** Estimated results of the impact of individual penalties on pollution emissions (excluding municipality samples).

	(1) lnsmoke	(2) lnsulfur	(3) lnsmoke	(4) lnsulfur
penalty	0.0452 (0.0161)	–0.0050 (0.0163)		
judicial			0.1213 (0.0201)	0.0576 (0.0239)
_cons	4.0596 (9.4505)	–50.4384^**^ (20.8726)	14.1857 (10.0924)	–45.5220^**^ (21.5606)
control variable	Yes	Yes	Yes	Yes
fixed time	Yes	Yes	Yes	Yes
fixed ind	Yes	Yes	Yes	Yes
F-test	116.52^***^	177.75^***^	175.67^***^	235.71^***^
R^2^	0.9251	0.8568	0.9297	0.8574
N	338	338	338	338

Robust standard errors in parentheses ^*^ p < 0.1,^**^p < 0.05,^***^p < 0.01

The results reported in Table 8 show that the estimated coefficients of the core explanatory variable D on judiciary are significantly positive at the 1 per cent level and the estimated coefficients on penalties do not pass the significance test after excluding the municipality samples. In addition, the estimated coefficients of the core explanatory variable D on lnsmoke and lnsulfur are both significantly negative. The results reported in Table 9 also show that the estimated coefficients on the effects of the core explanatory variables penalty and judicial on both lnsmoke and lnsulfur fail the significance test, suggesting that the intensity of individual penalties does not have a significant effect on the level of pollution emissions in the current period, even after excluding the sample of municipalities.

In summary, the results of the above robustness tests are consistent with the conclusions obtained from the benchmark regression.

## 5 Discussion

### 5.1 Emphasis on individual penalties alone leads to “punishment deals” betweenindividuals and local governments

The logic of punishment under the law is that individuals, deterred by the potential risk of punishment and the cost of punishment, choose to perform or refrain from performing certain behaviours as required by the law. Environmental laws often penalise individuals by requiring them to pay a fine or to be detained for a short period of time after committing an offence. The amount of the fine and the length of detention are based on the severity of the polluting behaviour of the individual. Ideally, the individual would be caught and punished by the government immediately after committing an act of pollution, and then the individual would stop polluting and refrain from doing so for fear of being punished again [29].

However, the ideal punishment deterrence often cannot be realized in reality. Because according to environmental law, the amount of punishment for environmental violations is often much lower than its benefits, resulting in individuals not afraid to accept legal punishment. For example, the water pollution prevention and control law (Article 85 of the Water Pollution Prevention Law of the People ’s Republic of China) imposes a fine of only 1 million yuan (140,000 US. dollars) on water pollution, usually a fine of 100,000 yuan (14,000 US. dollars). Or because the probability of environmental violations being discovered is not high, especially the common small-scale environmental violations in China, such as burning citrus. This kind of environmental pollution is mostly punctate and scattered, and it is difficult to be found in routine environmental inspections. In addition, large enterprises or industrial stakeholders are not afraid of being punished by environmental laws, but are willing to accept punishment to obtain the opportunity of pollution discharge. According to the revised environmental protection law in 2015, the fine for pollution caused by enterprises is generally calculated based on the direct economic losses caused by pollution, but it does not include the long-term losses caused by the decline of environmental quality, nor does it include the costs of ecological restoration (Article 59 of the Environmental Protection Law of the People ’s Republic of China). Moreover, its fines often have an upper limit. For example, prior to the 2015 amendment, the Air Pollution Prevention and Control Law (Article 48 of the 2000 Law of the People’s Republic of China on the Prevention and Control of Air Pollution) stipulated a maximum fine of CNY 300,000 (approximately USD 42,000). After the 2015 revision (Article 99 of the 2015 Law of the People’s Republic of China on the Prevention and Control of Air Pollution), the maximum fine was increased to CNY 1 million (approximately USD 140,000). Some scholars have proposed that the fine for environmental violations should be greatly increased, but this opinion is not suitable for the current situation of China ’s industry. A large number of small and medium-sized enterprises are in a state of loan survival, and the increased fines may directly lead to the bankruptcy of small and medium-sized enterprises. Large enterprises ( often state-owned enterprises ) can be exempted from major penalties by rent-seeking with local governments [30].

Therefore, in China’s environmental law practice over the years, a kind of “punishment deal” between individuals and the government has occurred repeatedly. The “punishment deal” is that the individual accepts the government’s punishment for violating the law after emitting pollution, but does not stop emitting pollution, and the government does not continue to punish the individual’s continuous polluting behaviours after completing the punishment once [31]. For the individual, to bear a punishment certainly caused a lot of losses, but the continuous pollution behaviour for their gains can completely make up for the losses suffered, or even more benefits.Taking Article 83 of the Water Pollution Prevention and Control Law as an example, illegal discharge of industrial sewage will be subject to a fine of 100,000 to 1 million. However, the production income of illegal discharge of industrial sewage by enterprises may be much higher than the upper limit of the fine of 1 million yuan. For example, in the “Zijin Mining Pollution Incident”, which shocked China, Zijin Mining gained profits exceeding one billion yuan by discharging pollutants. For details, see Liu Baijun, “Zijin Mining Trial Details Disclosed,” Legal Daily, February 14, 2011, page 008.Thus, penalties are not sufficient to deter individuals from stopping polluting. Moreover, China’s environmental laws have long adhered to the principle of “no more penalties for one thing, no more penalties for another,” which means that the government will not penalise an individual again for the same, ongoing pollution act [32]. As a result, the individual who receives the penalty also obtains the “right to discharge water in violation of the law” in disguise. The government, on the other hand, has fulfilled its legal responsibility under the environmental law by imposing the penalty. Both the individual and the government are satisfied with the end of the punishment, and at the same time acquiesce in and ignore the continuation of environmental pollution, which ultimately leads to the continuation or even further expansion of the polluted discharge.

### 5.2 Emphasis on individual punishment alone leads to misplaced responsibility onthe part of the government

Chinese lawmakers have also discovered the existence of “penalty trading”, so after the 2015 revision of the Environmental Protection Law, the Law changed the principle of “no more penalty for one incident” to the principle of “daily penalty”, which means that the government can impose new penalties for persistent pollutant emissions by the number of days after completing one penalty [33]. However, this does not mean that the government is willing to impose new penalties on individuals for persistent pollution emission behaviour. Because environmental laws emphasise penalties for individuals, the legal responsibility that the law places on the government is the responsibility to regulate and penalise individuals, not the responsibility to reduce pollution emissions [17]. This not only implies that the government has fulfilled its responsibility under the law when it has completed the first punishment of the individual, but also implies that the imposition of a new punishment on the individual does not bring the government new performance in fulfilling its responsibility. The reason is that whether it is “no more penalty” or “daily penalty”, it is the same individual’s same offence, which belongs to the same performance of the local government [34]. A new penalty imposed by the local government will not only cost more, but will also have no new benefits. Therefore, although the new Environmental Protection Law stipulates the principle of “daily penalties”, as long as “environmental laws emphasise individual penalties” remains unchanged, the government’s legal responsibility is limited to regulating and punishing individuals, and the “punishment deal” between individuals and the government is not changed. The “punishment deal” between individuals and the government will not be broken.

The existence of “penalty trading” is a good explanation of why punishing individuals does not necessarily reduce pollution emissions, i.e. it explains the absence of a correlation between the intensity of punishment and pollution emissions. This is not to deny the positive benefits of punishing individuals, but to say that it is wrong to fetishise individual punishment. China’s efforts to control pollution emissions through environmental laws in recent years have been effective, and there must be positive contributions from punishing individuals, but the increasing intensity of punishment is not a sufficient condition for reducing pollution emissions.

China ’s environmental law needs a lot of reforms to break the superstition of individual punishment and get rid of the misunderstanding of over-reliance on individual punishment. First of all, the environmental legal system focuses on post-punishment and ignores the importance of source control and prevention principles. In the current legal system, the punishment for environmental pollution is often after the occurrence of pollution, and the provisions on how to prevent environmental pollution and reduce environmental risks are relatively weak. In order to solve this problem, environmental law should pay more attention to the principle of prevention, through the development of more stringent environmental standards, improve the effectiveness of environmental impact assessment, strengthen environmental monitoring and early warning mechanisms and other measures to achieve effective prevention of environmental pollution.Secondly, environmental law has insufficient consideration for the special circumstances of SMEs. SMEs often lack sufficient financial and technical support to meet the requirements of environmental protection. In the event of environmental violations, high fines may pose a serious threat to the survival of enterprises. Therefore, environmental law should introduce cost and risk sharing mechanisms such as environmental insurance, disperse environmental risks through market-oriented means, reduce the burden on small and medium-sized enterprises, and reduce the impulse of small and medium-sized enterprises to take illegal risks. Thirdly, environmental law should carry out targeted special settings for large enterprises ( especially state-owned enterprises ), from the administrative system to the economic system to restrict the environmental pollution behavior of large enterprises. Break the abnormal dependence between large enterprises and local governments, and reposition large enterprises as ordinary subjects in environmental relations, rather than subjects enjoying special pollution rights.Thirdly, the environmental law is relatively simple in the way of punishment, mainly relying on economic means such as fines. This single punishment method may not be able to effectively deal with complex environmental violations. Environmental law should explore a variety of penalties and remedies, such as daily penalties, restorative penalties, ecological compensation, etc., in order to improve the adaptability and effectiveness of the law. Effectively link the law with the improvement of environmental quality.Finally, the environmental law is not clear enough to stipulate the environmental legal responsibility of local governments, which leads to the weak sense of responsibility of local governments in environmental protection. Environmental law should focus on local governments, clarify the responsibility of local governments in improving environmental quality, and strengthen the environmental responsibility of local governments through environmental protection target responsibility system and assessment and evaluation system, so as to reduce the space and possibility of regulatory capture and rent-seeking.

## 6 Conclusion

The Chinese government has long believed that environmental laws must be centred on individual penalties, and although this does not mean the exclusion of other elements (e.g., market regulation, education and publicity, etc.), it does mean that the Chinese government has invested more social costs in individual penalties, such as legal regulation, lawsuits, and the giving of penalties, and will continue to do so in the future [35]. Supporting this input strategy is the fact that in the last two decades China has intensified individual penalties and reduced pollution emissions. This seemingly de facto causal link allows the Chinese government to adhere to a more conservative form of governance in the global context of environmental law reform. Of course, this conservatism is only a tactical conservatism, a conservatism relative to the developed world, and the Chinese government has never neglected or slackened off in its environmental governance or in its efforts to solve environmental problems. However, the core issue that the Chinese government should consider is that purely individual punishment is not enough, and that scientific environmental governance should move away from the adversarial and managerial modes represented by individual punishment to a cooperative mode full of trust, commitment, and shared understanding, i.e., Collaborative Mode [36]. This shift must be based on a foundation that breaks the “myth” of individual punishment in Chinese environmental law.

This paper takes the revision of the 2015 Environmental Protection Law, one of the biggest environmental law events in China in recent years, as the node, and analyses the correlation between individual punishment intensity and the degree of pollution emission before and after the revision. This paper draws three conclusions: conclusion 1 is that the revision of the 2015 Environmental Protection Law partially enhanced the individual punishment intensity of environmental laws, conclusion 2 is that the revision of the 2015 Environmental Protection Law significantly reduced the degree of pollution emission in China, and conclusion 3 is that there is no correlation between the individual punishment intensity and the degree of pollution emission. Conclusions 1 and 2 provide an objective basis for the analysis of Conclusion 3, and it is not possible to discuss whether “individual penalties can necessarily reduce pollution emissions” if the intensity of individual penalties is not increased or the extent of pollution emissions is not reduced. Fortunately, China before and after the amendment of the Environmental Protection Law provides a perfect data sample for this discussion, and although hypothesis 1 is partially proved, it does not affect the testing of hypothesis 3 and the conclusion 3.

The core conclusion of this paper (Conclusion 3) dispels the “myth” of individual penalties in environmental law and demonstrates that there is no causal link between the fact that individual penalties have been tightened in China over the last two decades and the fact that the total amount of pollutant emissions has been decreasing, i.e., individual penalties do not necessarily reduce pollutant emissions. No one can deny the importance of individual penalties for environmental governance, but this only means that individual penalties are a necessary condition for reducing pollution emissions, and this paper demonstrates that individual penalties are not a sufficient condition for reducing pollution emissions.

Unlike the vast majority of quantitative studies that ultimately reach a positive relevance conclusion, the relevance conclusion of this paper is negative. However, this negative correlation conclusion is of great value as a basis for the Chinese government to change its environmental laws. On the basis of this conclusion, this paper suggests the following countermeasures. Firstly, environmental law needs to change the positioning of the government and find a role for it as a co-operator in addition to its punitive role. Specifically, the government not only needs to be responsible for regulating and punishing individuals, but also needs to be responsible for environmental information disclosure, environmental protection publicity and education, and providing appropriate institutional design and arrangements for public participation in environmental governance [37]. Secondly, environmental law needs to focus on the role of enterprises in environmental governance, to provide sustainable endogenous power for environmental governance [38]. Specifically, environmental laws need to promote the transformation of corporations from the traditional roles of regulated, regulated and passive law-abiding to active participants, self-regulators and active law-abiding, so as to achieve the maximisation of the utility of corporate autonomy and contractual governance. Finally, the environmental law needs to stimulate the synergistic participation of social organisations [39]. Specifically, it means that environmental law needs to increase the support and regulate the management of environmental social organisations, do a good job of organising public participation in environmental governance, and form a pluralistic and common governance pattern of environmental governance.

Although this paper successfully dispels the “myth” of individual punishment in environmental law and provides a theoretical basis for the Chinese government to change environmental law, it still has shortcomings. From the perspective of shifting from individual penalties to collaborative governance, this paper only analyses the correlation between individual penalties and pollution reduction as the first step of the shift. It does not deal with the subsequent studies, such as whether the co-operation between enterprises and the government in China actually reduces pollution emissions? Therefore, research on the correlation between Collaborative governance and pollution reduction will be the direction of subsequent research. At the same time, this should also be the focus of future research in China’s practical and academic circles.

## Data Availability

The relevant data sources involved in this study are three categories: per capita GDP, population density and patent authorization are derived from ‘China Statistical Yearbook’ (2009–2021), industrial smoke (powder) dust emissions and sulfur dioxide emissions are derived from ‘China Environmental Statistics Yearbook’ (2009–2021), and the number of environmental protection administrative cases and the number of environmental protection judicial cases are derived from the China Judicial Judgment Document Network. The following are the details. China Environment Statistical Yearbook (2009–2021) Access channels: https://navi.cnki.net/knavi/yearbooks/YHJSD/detail?uniplatform=NZKPT. How to obtain: It can be downloaded through this link by purchasing the yearbook or purchasing HowNet members. Industrial smoke (powder) dust emissions and sulfur dioxide emissions are derived from the fourth chapter of the yearbook ‘atmospheric environment’. China Statistical Yearbook (2009–2021) Access channels: https://navi.cnki.net/knavi/yearbooks/YINFN/detail?uniplatform=NZKPT. How to obtain: It can be downloaded through this link by purchasing the yearbook or purchasing HowNet members. The per capita GDP is derived from the third chapter of the yearbook ‘National Economic Accounting’, the population density is derived from the second chapter of the yearbook ‘Population’, and the patent authorization is derived from the 20th chapter of the yearbook ‘Science and Technology’. China Judicial Judgment Documents Network Access channels: https://wenshu.court.gov.cn/website/wenshu/181029CR4M5A62CH/index.html. How to obtain: After registering on the website, selecting administrative cases, inputting keywords such as ‘environmental protection’, ‘atmosphere’, ‘water’ and other keywords related to the environmental field, we can obtain statistics on the number of environmental protection administrative cases. Select criminal cases, enter keywords related to the environmental field such as ‘environmental protection’, ‘atmosphere’, ‘water’, etc., and obtain statistics on the number of environmental protection judicial cases. In order to facilitate editing and readers access to relevant data, we attach the manuscript’s minimal data to the attachment ‘other’, and save it in EXCEL format, which includes all the data used in the paper. The descriptive statistics of the data are as follows: Table 1 Descriptive Statistics of Data Variable N Mean SD Min Max lnsmoke 390 12.30 1.019 8.362 14.40 lnsulfur 390 12.28 1.454 6.215 14.95 lnpgdp 390 10.78 0.505 9.241 12.12 lntech 390 10.02 1.523 5.576 13.68 lnpop 390 5.463 1.292 2.043 8.282 lnpgdp2 390 116.4 10.90 85.39 147.0 judicial 390 0.580 0.907 0.000252 8.424 penalty 390 0.521 1.063 0.000850 9.701 d 390 0.538 0.499 0 1 All regression results in the text are calculated using stata software. The commands used are as follows:

encode province,gen(province1)
reg judicial d i.province1,r
est store m1
reg penalty d i.province1,r
est store m2
reg judicial d lnpgdp lnpgdp2 lntech lnpop i.province1,r
est store m3
reg penalty d lnpgdp lnpgdp2 lntech lnpop i.province1,r
est store m4
esttab m1 m2 m3 m4 using table2.rtf, replace compress nogap b(4) se(4) scalar(N r2) star(* 0.1 ** 0.05 *** 0.01) obslast indicate( "province=*province1*")
reg lnsmoke d i.province1,r
est store m1
reg lnsulfur d i.province1,r
est store m2
reg lnsmoke d lnpgdp lnpgdp2 lntech lnpop i.province1,r
est store m3
reg lnsulfur d lnpgdp lnpgdp2 lntech lnpop i.province1,r
est store m4
esttab m1 m2 m3 m4 using table3.rtf, replace compress nogap b(4) se(4) scalar(N r2) star(* 0.1 ** 0.05 *** 0.01) obslast indicate( “province=*province1*”)
reg lnsmoke penalty lnpgdp lnpgdp2 lntech lnpop i.year i.province1,r
est store m1
reg lnsulfur penalty lnpgdp lnpgdp2 lntech lnpop i.year i.province1,r
est store m2
reg lnsmoke judicial lnpgdp lnpgdp2 lntech lnpop i.year i.province1,r
est store m3
reg lnsulfur judicial lnpgdp lnpgdp2 lntech lnpop i.year i.province1,r
est store m4 esttab m1 m2 m3 m4 using table4.rtf, replace compress nogap b(4) se(4) scalar(N r2) star(* 0.1 ** 0.05 *** 0.01) obslast indicate("year=*year*" "province=*province1*")
xtset province1 year reg judicial l.d lnpgdp lnpgdp2 lntech lnpop i.province1,r
est store m1
reg penalty l.d lnpgdp lnpgdp2 lntech lnpop i.province1,r
est store m2
reg lnsmoke l.d lnpgdp lnpgdp2 lntech lnpop i.province1,r
est store m3
reg lnsulfur l.d lnpgdp lnpgdp2 lntech lnpop i.province1,r
est store m4
esttab m1 m2 m3 m4 using table5.rtf, replace compress nogap b(4) se(4) scalar(N r2) star(* 0.1 ** 0.05 *** 0.01) obslast indicate( "province=*province1*")
reg lnsmoke l.penalty lnpgdp lnpgdp2 lntech lnpop i.year i.province1,r
est store m1
reg lnsulfur l.penalty lnpgdp lnpgdp2 lntech lnpop i.year i.province1,r
est store m2
reg lnsmoke l.judicial lnpgdp lnpgdp2 lntech lnpop i.year i.province1,r
est store m3
reg lnsulfur l.judicial lnpgdp lnpgdp2 lntech lnpop i.year i.province1,r
est store m4
esttab m1 m2 m3 m4 using table6.rtf, replace compress nogap b(4) se(4) scalar(N r2) star(* 0.1 ** 0.05 *** 0.01) obslast indicate( "province=*province1*")
reg judicial d lnpgdp lnpgdp2 lntech lnpop i.province1 if province1 = 1& province1=4& province1=7& province1=27,r
est store m1
reg penalty d lnpgdp lnpgdp2 lntech lnpop i.province1 if province1 =1& province1=4& province1=7& province1=27,r
est store m2
reg lnsmoke d lnpgdp lnpgdp2 lntech lnpop i.province1 if province1 =1& province1=4& province1=7& province1=27,r
est store m3
reg lnsulfur d lnpgdp lnpgdp2 lntech lnpop i.province1 if province1 =1& province1=4& province1=7& province1=27,r
est store m4
esttab m1 m2 m3 m4 using table7.rtf, replace compress nogap b(4) se(4) scalar(N r2) star (* 0.1 ** 0.05 *** 0.01) obslast indicate (“province=*province1*")
reg lnsmoke penalty lnpgdp lnpgdp2 lntech lnpop i.year i.province1 if province1 =1& province1=4& province1=7& province1=27,r
est store m1
reg lnsulfur penalty lnpgdp lnpgdp2 lntech lnpop i.year i.province1 if province1=1& province1=4& province1=7& province1=27,r
est store m2
reg lnsmoke judicial lnpgdp lnpgdp2 lntech lnpop i.year i.province1 if province1=1& province1=4& province1=7& province1=27,r
est store m3
reg lnsulfur judicial lnpgdp lnpgdp2 lntech lnpop i.year i.province1 if province1=1& province1=4& province1=7& province1 =27,r
est store m4 esttab m1 m2 m3 m4 using table8.rtf, replace compress nogap b(4) se(4) scalar(N r2) star(* 0.1 ** 0.05 *** 0.01) obslast indicate( "province=*province1*").

## References

[1] Tu Y, Peng B, Elahi E, Wu W. Initiator or intermediary? A case study on network relation of environmental regulatory capture in China. Int J Environ Res Public Health 2020;17(24):9152. doi: 10.3390/ijerph17249152 33302337 PMC7762369

[2] He B. How does capture affect environmental regulation? - a mixed study. Publ Admin Policy Rev. 2023;12(04):113–28.

[3] Cao C, Li Y. Economic analysis of rent-seeking in environmental governance. Environ Protect Circular Econ. 2011;31(02):19–22.

[4] Coase R. The problem of social cost. J Law Econ. 2013;56(4):837–77.

[5] Libecap GD. The tragedy of the commons: property rights and markets as solutions to resource and environmental problems. Austral J Agricult Resource Econ. 2009;53(1):1–17.

[6] Deryugina T, Moore F, Tol RSJ. Environmental applications of the coase theorem. Environ Sci Policy. 2021;120(5):81–8.

[7] Scott RJ, Merton ERK. When the going gets tough, the goal-committed get going: overcoming the transaction costs of inter-agency collaborative governance. Publ Manag Rev. 2021;2(2):1–24.

[8] An R, Sang T. The guarantee mechanism of China’s environmental protection strategy from the perspective of global environmental governance-focusing on the punishment of environmental pollution crime in China. Int J Environ Res Public Health 2022;19(22):14745. doi: 10.3390/ijerph192214745 36429464 PMC9690176

[9] L. 9, Zhongmei W. Seventy years of environmental rule of law in china: From history to future. Chinese Law Rev. 2019;5(5):102–23.

[10] Yu C, Morotomi T. The effect of the revision and implementation for environmental protection law on ambient air quality in China. J Environ Manage. 2022;306:114437. doi: 10.1016/j.jenvman.2022.114437 34998089

[11] Zhuoyue W, Bin W, Yuheng Z, Rong H, Qingdan Y. Forty years of development of environmental rule of law in China: achievements and experience. Environ Sustain Develop. 2018;43(06):5–10.

[12] Zhong S, Xiong Y, Xiang G. Environmental regulation benefits for whom? Heterogeneous effects of the intensity of the environmental regulation on employment in China. J Environ Manage. 2021;281:111877. doi: 10.1016/j.jenvman.2020.111877 33370676

[13] Clark H, Pinkovskiy M, Sala-I-Martin X. China’s GDP growth may be understated. China Econ Rev (2018)

[14] Fang J. Environmental law, environmental policy stringency, and development of environmental technologies in China. Environ Sci Pollut Res Int 2023;30(45):101234–49. doi: 10.1007/s11356-023-29023-5 37648917

[15] Z. Y. Adhere to the strictest system and the strictest rule of law to protect the ecological environment. Pioneer. 2018;9(9).

[16] Hainan L. Strengthening pollution control according to law and promoting environmental compliance to improve. China Econ Times (2002).

[17] Yuan J. Reflection and reconstruction of government environmental legal responsibility. J China Univ Geosci (Soc Sci Edn). 2020;20(2):33–40.

[18] Yuan J. To explore and construct the environmental quality target system of the simultaneous presence of the central and local governments – taking the dispute over the normalization of environmental protection inspectors as the starting point. Politic Legal Theory. 2021;02:140–9.

[19] Song H. 75 years of China’s environmental policy: historical evolution, driving force and future trend. J Fujian Norm Univ (Philos Soc Sci Edn). 2024;05:91–104.

[20] Zhu D. China’s major environmental case review: Zi 7 gold mining water pollution case. Environ Protect Circul Econ. 2013;33(02):28–31.

[21] Jiwen C. New environmental protection law: toughest in history but most difficult to enforce. Environ Protect. 2014;42(10):23–8.

[22] Zhang B, Cao C, Gu J, Liu T. A new environmental protection law, many old problems? challenges to environmental governance in china. J Environ Law. 2016;28(2):325–35.

[23] Z. Y, Z. Y. Impact of environmental regulation on ecological efficiency under the background of new environmental protection law. J Environ Protect Ecol. 2020:6(6);21.

[24] York R, Rosa EA, Dietz Stirpat T. ipat and impact: analytic tools for unpacking the driving forces of environmental impacts. Ecol Econ. 2003;3:351–65. doi: 10.1234/example.doi

[25] Wani SA,. Environmental justice in India - an analysis of role of National Green Tribunal. J Univ Inst Stud. 2022;16(1):131–48.

[26] Dinda S. Environmental Kuznets curve hypothesis: a survey. Ecol Econ. 2004;49(4):431–55.

[27] Liu J, Li S, Ouyang Z, Tam C, Chen X. Ecological and socioeconomic effects of China’s policies for ecosystem services. Proc Natl Acad Sci U S A 2008;105(28):9477–82. doi: 10.1073/pnas.0706436105 18621700 PMC2474515

[28] People’s procuratorates of provinces, autonomous regions and municipalities directly under the central government and military procuratorate. China Law Yearbook English Edition. 2003.

[29] Shimshack JP. The economics of environmental monitoring and enforcement. Annu Rev Resour Econ 2014;6(1):339–60. doi: 10.1146/annurev-resource-091912-151821

[30] Ren T, Xiao Y. Environmental penalties and corporate cash holdings - based on empirical evidence from Chinese companies. Audit Econ Res. 2023;38(02):67–77.

[31] Xiangmin X. Environmental quality goalism: Reflections on the objectives of direct regulation of environmental law. Chinese Law. 2015;6:116–35.

[32] Fengmiao H. Analysis on the application of the principle of “no punishment for one thing’’ in environmental administrative punishment Poyang Lake J. 2019;(2):88–94+127.

[33] Hu K, Li D, Shi D. Environmental regulation and energy efficiency: evidence from daily penalty policy in China. J Regulat Econ. 2023;63(1–2):1–29.

[34] Bu C, Shi D. The emission reduction effect of daily penalty policy on firms. J Environ Manage. 2021;294:112922. doi: 10.1016/j.jenvman.2021.112922 34102466

[35] Wu H-Q, Shi Y, Xia Q, et al. Effectiveness of the policy of circular economy in China: a DEA-based analysis for the period of 11th five-year plan. Resources Conserv Recycling. 2014;83:163–75.

[36] Debra J, Davidson S. Understanding environmental governance. Organiz Environ. 2016.

[37] Dong L, Wang Z, Zhou Y. Public participation and the effect of environmental governance in china: a systematic review and meta-analysis. Sustainability. 2023:15(5);4442.

[38] Chen R, He X, Bidabadi FS. Corporate environmental compliance in china: from social responsibility to soft law. Sustainability. 2023:15(3);2379.

[39] Lit YW. Disembedding lawful activism in contemporary china: The confrontational politics of a green NGO’s legal mobilization. China Inf. 2018;32:224–43.

